# Chicago Society of Physicians and Surgeons

**Published:** 1874-12

**Authors:** Ralph E. Starkweather


***Chicago Society of Physicians and Surgeons.*** *Transactions at Regular Meeting, Monday, Oct.* 12, 1874. Reported by RALPH E. STARKWEATHER, M.D.

Dr. Bartlett, President, in the chair. After the usual preliminaries. Dr. Steele read a paper, which was a report of a case of pneumatic aspiration of the pericardial sac, by Dr. Johnson, at the Cook County Hospital.

The following is a synopsis of the report:

The patient was a German laborer, forty-five years of age, and in good health previous to August last. Early in that month, while employed in a saloon, he went into an ice cellar, when sweating profusely, to cool off. Several slight chills followed, accompanied by severe cramping pains in back and left side, over the region of the heart. A deep inspiration aggravated the pain; there was no cough; appetite was impaired; bowels costive; breathing labored. At the end of a month, he had convalesced, and began work as a cook. He exposed himself again to sudden changes of temperature, and slept in a damp basement. Another attack similar to one above mentioned set in, with cough and expectoration of white frothy sputa, the breathing becoming more frequent and labored. Has been a moderate drinker for a number of years; family history devoid of tuberculous taint; never had syphilis, nor rheumatism. Condition on admission, September 22, 1874: Patient is a large, well nourished man; decubitus on left side, shoulders elevated, thigh flexed on abdomen. The breathing is hurried and laborious; face anxious and cyanotic; skin cool and moist. Pulse 132, small, thready and irregular; respiration 28 to the minute; temperature, little less than normal.

PHYSICAL EXAMINATION.

*Inspection.*—There is a bulging of left chest and precordial region; epigastric protrusion; partial obliteration of left intercostal spaces, and decided loss of motion in left chest. Heart communicates no thoracic shock.

*Palpation.*—There is an absence of vocal fremitus in left chest, anteriorly, while, posteriorly, the fremitus is exaggerated.

*Percussion.*—There is complete dullness in left chest anteriorly over a somewhat irregular triangle, of which the base would correspond with a transverse line drawn from left axillary region three inches below left nipple, to a point two inches to right of the xiphoid appendix. The apex of the heart is at a point about five inches above, and one and a half inches to right of left nipple. The dullness was but slightly altered by postural changes.

In the right chest, the resonance was slightly increased.

*Auscultation.*—Below the third rib there was absence of all lung sounds of left lung. * * * * Posteriorly occasional fine crepitant rales, and friction sounds. In the cardiac region, there were no heart sounds. There were a few to and fro pericardial friction sounds.

The diagnosis was, acute pericarditis, with a large serous effusion.

*The operation* of pneumatic aspiration was done by Dr. H. A. Johnson. The patient being placed in a sitting posture, a fine canula, connected with a vacuum, was introduced into the fifth intercostal space, two inches to the left of the sternum, and carefully carried inwards, upwards and backwards, till it had penetrated the thoracic walls about two inches; a few drops of bloody serum escaped; about one ounce was removed, and the canula was withdrawn. Probably the canula entered a little pouch or pocket formed at the base of the pericardium by plastic lymph, and did not enter the main collection of fluid.

The immediate effect of the aspiration was to quicken and strengthen the circulation as determined by the radial pulse. The heart continued intermittent.

September 24. The patient felt better and could breathe more easily. Four days later a pleuro-pneumonia of the left chest set in, and increased the gravity of the symptoms. September 30. After a consultation of the attending physicians, it was determined to again perform aspiration as a *dernier resort.* A fine canula was introduced, near the point of previous puncture, in a similar direction and a little higher than before, till the point had passed into the chest about two inches, when on turning the stop cock the skillful operator was rewarded by seeing a full stream of bloody serum flowing from the pericardial sac into the receiving bottle. Eleven and one-half ounces were withdrawn, without the occurrence of a single unpleasant symptom.

The immediate effect of the operation was to relieve the turgescence of the superficial vessels, and steady and strengthen the heart's action; the breathing became easier, and the apex beat of the heart could be seen, for the first time, striking a little to the left and higher than normal; the cardiac sounds were distinctly heard. The bulging of the intercostal spaces was not so marked. Half an hour after the operation, the pulse was one hundred and thirty-two (132); respiration, twenty-eight (28) per minute.

October 1. Pneumonia more extensive, extremities cold; radial pulse imperceptible; general cyanosis; delirium and death 1 A.M., October 2d.

Dr. Steele then exhibited to the Society the heart of this patient, and demonstrated the points of entrance of the needle into the sac. He gave the following details of the post mortem examination made on the day of the death. The entire body was cyanotic—the pericardium was immensely distended by fluid, occupying nearly the whole anterior portion of the left chest, overlapping the right lung for two or three inches. Diaphragm depressed; moderate pleuritic effusion in both chests. On opening the sac, it was found much thickened and congested, and contained about one hundred ounces of bloody serum. The point of the second puncture was readily seen; that of the first puncture had closed by adhesive inflammation. The heart was hypertrophied, dilated, apparently inflamed; its right side distended with venous coagula; the entire surface of the heart was covered by a tenacious coagulated lymph, arranged in segregated layers, varying in depth from one-half to one inch, so firmly adherent to the walls of the heart as to be with difficulty removed. The pericardium was adherent to the heart, around its base, by means of this plastic deposit. Left lung throughout was in the stage of red hepatization—quite friable, and firmly adherent to chest walls by recent pleuritic adhesions. The right lung was congested; kidneys healthy. * * * *

In commenting upon the case, Dr. Steele said that no heart sounds were heard till after the second tapping; but that the valves were probably not diseased; the heart itself was not weighed, but the weight of heart and its sac was probably four pounds. The patient when in health weighed one hundred and seventy pounds, and was five feet eleven inches in height. Before operating, the patient was advised fully as to the operation, and that it was only expected to give temporary relief; that in itself the operation was not curative. A needle, a little larger than the one used in a hypodermic syringe, was employed.

In reply to an inquiry by Dr. Merriman, it was stated that the patient had never had rheumatism, but had been a large healthy man, until three months ago.

Dr. Hamill expressed some doubts as to the advantage of aspiration, or its expediency. If there is absorption or removal of the fluid, may there not then be such adhesions as to hasten death?

Dr. F. H. Davis asked whether there was any effusion into the pleural cavities, and Dr. Steele said that there were twenty-five to thirty ounces in each cavity, the latter amount in the right chest.

Dr. F. H. Davis. It seems as though there might be one chief danger in this operation, the liability of the puncture to allow a dribbling of the fluid into the pleural sac, and produce a pleurisy or perhaps pneumonia; of course, the character of the fluid effused would determine this question—if it were purulent, the result might be different.

One of the members called attention to the fact that this patient, from the history given us, seems to have had a *primary* and *idiopathic pericarditis*, which is certainly very rare; at the time of death, it was not solitary, for there was double pleurisy and a pneumonia. In the morbid specimen, he saw no deposit of lymph upon the parietal serous surface; the cardiac portion of the sac was everywhere thickly covered by the exudation. He then alluded to the gravity and rarity of the operation, and its success.

Dr. Hyde. So far as known, this operation is the first one ever done in Chicago, and must have required the courage if not the audacity which that other great physician (Trousseau) exhibited, who boldly plunged the needle into the pericardial fluid, long before Dieulafoy had established the present method and processes.

A discussion then ensued between Drs. Simon, Merriman and Hyde, as to the probable reason why the fluid that had been drawn off was bloody, and also as to the diseases men exhibit who follow the ice business.

Dr. F. H. Davis read several interesting reports of cases in his clinic. One was a case of nasal catarrh. Another a case of functional disturbance of the heart, due to nervous shock caused by a fall. A third case was one of congenital malposition of the heart and absence of the sternum in a young boy, with tubercles in the apex of either lung.

The President reported a case of cerebro spinal meningitis, which proved fatal, in which were numerous curious features simulating hydrophobia. The discussion of this subject, including rabies, was animated and exhaustive.

Dr. Hamill reported a case of abortion with retained placenta, in which he used full doses of ergot for twelve hours, without effect. He then exhibited the fluid extract of *actea racemosa*, forty drops every two hours; the secundines were expelled in six hours; the medicine seemed to act on the body of the uterus, producing tonic contractions. He preferred it rather than ergot. He had been called to the case two days after the abortion took place.

Several members related cases much resembling the one given with like experience, and expressed the opinion that the use of ergot in the third stage of parturition was, at least, very questionable, and inexpedient.

Dr. Merriman was appointed by the President to prepare a paper to be read at the next meeting, upon the management of the third stage of labor.

At a late hour the Society adjourned.

*Transactions at Regular Meeting, October* 26, 1874.

Dr. Bartlett, President, in the chair.

After the reading of the minutes of the previous meeting, the name of Dr. R. L. Leonard was favorably reported to the Society by the Board of Censors and received a unanimous election. The Society listened to the reading of a translation from the German, of Drs. Kundrat, of Vienna, and Englemann, of St. Louis, on the Microscopical Appearances of the Uterine Mucous Membrane, made and read by Dr. Von Mansfelde. At its conclusion, a vote of thanks was carried, but owing to the length of time occupied by the reading of the paper, upwards of two hours, its discussion was deferred.

*Transactions at Regular Meeting, November* 9, 1874.

Dr. Bartlett, President, in the chair.

After the usual preliminaries, a motion of suspension of the rules was carried, in order to receive a communication from a committee of the Chicago Medical Society in regard to a joint donation of money, in temporary support of the widow and young son of a physician who had died soon after the great calamity of 1871. The subject was referred to a committee, to report at the next meeting.

Dr. Etheridge, a member of the Section on Materia Medica and Therapeutics, presented a very full report, including translations and various interesting items from recent publications.

Attention was first called to the new Brazilian sudorific and sialagogue, *Jaborandi.*

This is a South American product; the leaves and small twigs are the parts of the plant used. The leaves have an odor and a bitter taste, but do not appear to have any alkaloids. Sixty to ninety grains of the leaves and twigs may be infused in a cup of water. "When taken, a drenching perspiration, or, more properly, sweating, lasting four or five hours, necessitating several changes of clothes, will follow. At the same time an abundant salivary and bronchial secretion supervenes, which may even exceed two pints in quantity."

The amyl hydride was exhibited to the Society, and was considered to be innocuous when applied to the skin or mucous membranes. If swallowed, it is quickly converted into vapor. It is remarkable solvent. A solution containing one scruple of iodine to the ounce of amyl hydride is very simple, painless and effective when applied to bad smelling sores, ulcers, wounds, and to cancer. A very quick disinfectant.

Common oils, spermaceti, and other similar oily substances, make ready solutions in amyl hydride. Burns may be treated with success by using such a solution.

After explaining the mode of making the nitrite of amyl, and exhibiting a sample thereof, it was described as diminishing arterial blood tension, as though the vaso-motor nerve supply were paralyzed or cut off. It is a remedy indicated in all cases of cerebral anæmia; in epilepsy, when due to that cause. If employed for this purpose, it should be given at the commencement of the attack, by inhalation of three or four drops placed in a small vial. In syncope, hemicrania and chloroform narcosis, amyl has been of benefit.

A case reported by Dr. Jenks was cited, of puerperal eclampsia, successfully treated by the nitrite, before and after labor, administering two or three drops by inhalation.

Passing next to the subject of carbolic acid hypodermic injections, several cases lately reported were cited, in which results very surprising and satisfactory had been attained. In all forms of parenchymatous inflammation it is an active antiphlogistic. The insertion should be made where the lymphatics bring the acid in direct contact with the inflamed tissue. The strength should be two per cent. of pure acid, in water. A solution of one per cent., the injections being repeated four or six times, afforded relief in two cases of erysipelas.

*Fissures in Ano, treated with Iodoform.*—As a local anæsthetic, it allays the spasm of the sphincter during defecation, while it favors cicatrization by neutralizing the irritating effects of fæcal matters which may remain on the ulcerated surface. It is used as an ointment, one part to three of lard, applied (twice daily) on a small piece of charpie. Cure may be expected to follow within twenty days.

*Bromide of Potassium.*—This salt has been employed by Dr. Bernard, in Algeria, in the treatment of engorgements of the spleen, due to intermittent fever. Complete resolution may be secured, the treatment being rarely prolonged beyond thirty days. Hypertrophies of the liver yield just as readily, or at least are very much improved. The dose of the bromide is fifteen grains daily, in one potion, in an infusion of orange leaves, etc., for fifteen to twenty days. In one case, however, during ten days, forty-five grains were given daily, in one potion, followed by cure.

An article by Dr. Chauppe, on "The Experimental Study of the Action of Ipecac," translated by Dr. Etheridge, was then read. It discussed the action of ipecac in arresting diarrhœa. At a former meeting an article upon its employment in phthisical sweats had been read.

No attempt will be made to give an abstract of this most valuable paper, partly on account of its length, but chiefly because the argument and details would be injured by being at all abbreviated.

Dr. Simons reported, verbally, a case of compound comminuted fracture of the right parietal bone, complicated by the protrusion of at least a half ounce of cerebral matter. The patient was a boy, of the age of three years. Half an ounce of the brain matter and a piece of bone one-half by three-fourths of an inch in size, were removed. The depressed bone was elevated; the lips of the scalp wound were united by sutures. At no time has there been loss of sense, sensation, intellection or motion. The boy is doing well, at the present time, four days after accident.

Dr. Hollister narrated a similar case that had occurred in his practice a number of years ago. The patient was a boy, twelve years of age, who had been kicked by a horse. There was a crucial fracture of the parietal bone near the frontal bone; the meninges were lacerated; the brain substance broken up, of which six drachms were lost. A piece of the parietal bone, one and one-half by one and three-fourths inches, was removed. The wound was closed, and compresses applied. Recovery was perfect. He never lost consciousness, nor was there failure of the mental powers. He was, however, pretty irritable in temper for a short time.

The motion to reconsider the vote adopting the report and resolutions offered at the last meeting, concerning the relations between physicians and pharmacists, was taken from the table, and, on motion, was laid upon the table indefinitely; thus sustaining the endorsement of them made by the previous meeting.

The Society then adjourned.

